# The Use of Intravenous Zoledronate May Reduce Retear Rate after Rotator Cuff Repair in Older Female Patients with Osteoporosis: A First In-Human Prospective Study

**DOI:** 10.3390/jcm11030836

**Published:** 2022-02-05

**Authors:** Jae-Hoo Lee, Jae-Young Yoon, Yong-Beom Lee

**Affiliations:** Department of Orthopaedic Surgery, Hallym University Sacred Heart Hospital, Medical College of Hallym University, Chuncheon 24252, Korea; holleewho@gmail.com (J.-H.L.); yoonjy5@naver.com (J.-Y.Y.)

**Keywords:** osteoporosis, rotator cuff repair, bisphosphonate, zoledronate, retear

## Abstract

The objective of this study was to demonstrate the effect of intravenous (IV) zoledronate administration on rotator cuff healing, retear rate, and clinical outcomes in osteoporotic patients who underwent arthroscopic rotator cuff repair (ARCR) compared with patients with normal bone densities. In this prospective nonrandomized comparative study with propensity score matching, 30 patients who were postoperatively administered IV zoledronate (5 mg) were enrolled as the study group. The control group was matched using 1-to-2 propensity score matching. Radiologic and functional outcomes were evaluated 6 months after surgery. The functional scores in both groups exhibited significant improvement 6 months after surgery. Compared with Group 1 (osteoporosis with IV zoledronate injection) Group 2 (normal bone density) showed significant improvement in their University of California, Los Angeles (UCLA) shoulder score and Constant Shoulder Score (CSS) at 6 months postoperatively. The range of motion improved in both groups at 6 months after surgery. The retear rates according to Sugaya’s classification (IV and V) were 13.3% (4 of 30 patients) and 25% (15 of 60 patients) in Groups 1 and 2, respectively, which established a non-inferiority of Group 1 to the control group. The retear pattern according to Rhee’s classification in Group 1 was type I in all cases, whereas eight cases of type I and seven cases of type II patterns were observed in Group 2, which was statistically insignificant between the groups. In conclusion, anti-osteoporotic drug use is beneficial for patients with osteoporosis to reduce the failure rate after an ARCR of length > 2 cm, especially in older female patients. Moreover, thorough scrutiny is required to detect osteoporosis in patients with rotator cuff tears, especially in female patients.

## 1. Introduction

Rotator cuff tears are common pathologic glenohumeral joint conditions frequently encountered by orthopedic surgeons. The gold standard mitigation for this condition is surgical repair because the spontaneous healing of these tears is not feasible. Recently, despite substantial efforts to promote healing after surgical repair, the retear rate of repaired rotator cuff tears < 2 cm in size was reported to be < 10%, while that of rotator cuff tears > 2 cm was up to 94% [1,2,3].

Although surgeries repair the torn rotator cuff in place, bone losses at the insertion sites do not tend to completely recover unassisted [4]. Hence, osteoporosis is a substantial risk factor for rotator cuff tears and increased retear rates [5]. Older people are usually the most vulnerable to this condition. Osteoporosis hinders bone–tendon healing at the repair site [6] and severely affects the pull-out strength of an anchor, leading to anchor loosening and eventual bone pull-out before bone–tendon healing is achieved [7]. Consequently, osteoporosis in patients with rotator cuff tears is challenging for surgeons.

Generally, bisphosphonates are known as anti-osteoclastic agents that support bone mineral density (BMD) by inhibiting the activation and maturation of osteoclast precursor cells into osteoclasts; in addition, they are effective in preventing osteoblast and osteocyte apoptosis [8]. Zoledronic acid is an anti-osteoporotic drug with high compliance because it is administered intravenously (IV) once a year. Although zoledronic acid is washed out of the body after IV infusion, it remains active for a year by adhering to bone surfaces immediately after incorporating it into the bone tissue. Several authors have reported on the effect of using bisphosphonates, such as zoledronic acid, on the promotion of BMD and bone integrity at the bone–tendon junction [9,10,11,12,13].

This is the first study investigating the effect of an IV zoledronate on rotator cuff healing in human. This study hypothesized that the administration of IV zoledronic acid improves functional outcomes and diminishes retear rate after arthroscopic rotator cuff repair (ARCR) over population with normal bone densities. Accordingly, the objective of this study was to demonstrate the effect of IV zoledronate administration on rotator cuff healing, retear rate, and clinical outcomes in older patients with osteoporosis who underwent ARCR compared with patients with normal bone densities.

## 2. Materials and Methods

The current study was a prospective nonrandomized comparative study with propensity score matching adopted for the control group. The study protocol was approved by the Institutional Review Board of Hallym University Sacred-Heart Hospital (No. HALLYM2019-04-017-001), and informed consent was obtained from all patients in the treatment group.

### 2.1. Study Design

We designed the study to compare osteoporotic patients who were treated with IV zoledronate with the control group assigned using propensity score matching among whom had normal bone density in terms of retear rate after arthroscopic rotator cuff repair.

We prospectively enrolled patients between February 2019 and April 2021. The inclusion criteria were as follows: (1) full-thickness rotator cuff tear > 2 cm in size, as verified by oblique-coronal pre-operative magnetic resonance imaging (MRI); (2) age > 60 years; (3) BMD with T-score < −2.5; (4) post-operative administration of anti-osteoporotic agent (5-mg IV zoledronic acid (Daewoong, Seoul, Korea); and (5) provision of informed consent.

The exclusion criteria were as follows: (1) follow-up loss before 6 months (*n* = 6) (2) no MRI evaluation before surgery and 6 months after surgery to evaluate rotator cuff integrity; (3) additional repair techniques other than transosseous equivalent repair due to irreparability, including superior capsular reconstruction, patch augmentation, and partial repair (*n* = 12); (4) history of surgical treatment on the affected side of the arm and shoulder (*n* = 1); (5) infectious, autoimmune, or systemic skeletal disease; (6) rotator cuff arthropathy, moderate-to-severe osteoarthritis, or rheumatoid arthritis; (7) no MRI due to implants, such as pacemakers; (6) no informed consent provided; (8) prior administration of any anti-osteoporotic agent, except vitamin D or calcium supplement; and (9) contraindications to anti-osteoporotic drugs (hypersensitivity, hypocalcemia, pregnancy, and kidney disorder with creatinine clearance < 35 mL/min, *n* = 2).

Chung et al. reported a significantly higher retear rate in osteoporotic patients with BMD under −2.5 (41.7%) compared with patients with BMD over −2.5 (13.2%; *p* < 0.001) [5]. Via power analysis, the minimum sample sizes of more than 14 patients for 1-to-2 matching was required for a power of 80% with a type I error of 0.05.

### 2.2. Group Assignment

The 49 patients who met the inclusion criteria were enrolled in the study. During surgery, twelve patients who required additional patch augmentation procedures (*n* = 7), superior capsular reconstructions (*n* = 3), and revision (*n* = 2) surgeries on the same shoulder were excluded. One patient who had an experience of surgical treatment on the same shoulder was also excluded. Additionally, six patients were lost to follow up. Accordingly, data analysis was performed for 30 patients (Group 1).

Regarding propensity score matching, between January 2018 and April 2021, 576 consecutive patients underwent surgical treatments for rotator cuff tears at our institution. Among them, except for the number of the control group, 318 patients had rotator cuff tears > 2 cm, were over 60, and were followed up for 6 months after surgery. Patients who underwent patch augmentation (*n* = 18), superior capsular reconstruction (*n* = 12), revision surgery (*n* = 10), and patients with osteoporosis/osteopenia (*n* = 42) were excluded.

**Figure 1:**
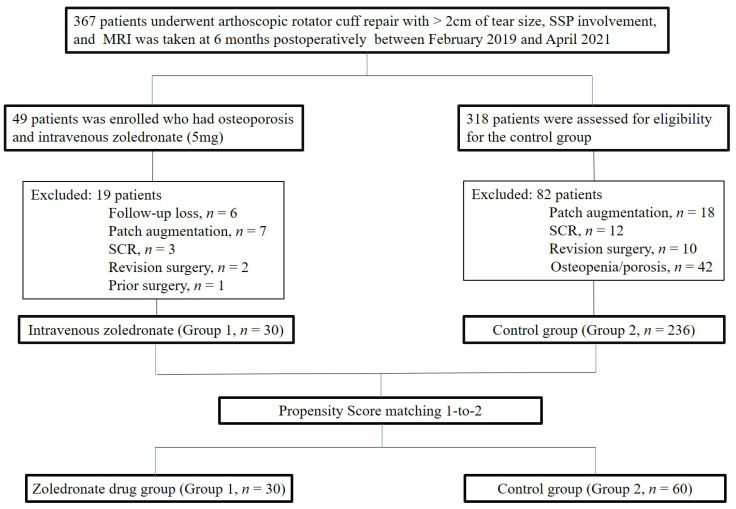
Flow diagram illustrating included and excluded patients.

K-nearest neighbor matching was adopted with variables, including age, sex, involved muscles (supraspinatus, supraspinatus and subscapularis, supraspinatus and infraspinatus, or all 3 muscles), preoperative fatty degeneration of the supraspinatus, infraspinatus, subscapularis, global fatty degeneration index, tear size of the tendon measured on MRI, history of smoking (regular smoking within a year or not) or trauma (only falling down and no patient with major traumatic events), and regular exercise (exercise more than 2 days in a week or less). Finally, propensity score matching was performed for the remaining 236 patients for a control group. Owing to the distinctively slanted distribution of age in Group 1, 1-to-2 matching was performed and 60 patients were assigned to Group 2. Figure 1 illustrates the selection algorithm adopted in this study.

### 2.3. Outcome Assessment

To assess clinical outcomes, the simple shoulder test (SST) score [14], University of California, Los Angeles (UCLA) shoulder score [15], American Shoulder and Elbow Surgeons (ASES) score [16], Constant Shoulder Score (CSS) [17], and shoulder range of motion (ROM) were obtained in all patients before surgery and 6 months after surgery. During each visit, a research assistant measured the functional scores. A goniometer was utilized to measure ROM, including forward flexion and internal rotation. Internal rotation was inferred to as the highest vertebral level at which the tip of the thumb could reach. The vertebrae were numbered in series, from below the sacrum (0) to the fourth thoracic vertebra (14). ROM was measured by the same research assistant. BMD was preoperatively checked for all patients who was menopausal or aged above 60 years for women or above 75 years for men using dual-energy X-ray absorptiometry (Lunar Prodigy Advance, Encore, GE Medical System, Milwaukee, WI, USA). The lowest T-scores of the lumbar spine (L1–4) and proximal femur were recorded, excluding the value of ward area of the femur. A T-score of < −2.5 was defined as “osteoporosis”.

### 2.4. Imaging Evaluation

All patients underwent MRI preoperatively and at 6 months after surgery [18]. All shoulder MRI evaluations were performed using a 3.0-T scanner (Signa HDx; GE Healthcare, Milwaukee, WI, USA) with a dedicated shoulder coil. Some of the preoperative MRI scans were taken at other hospitals before the first visit to our clinic. Patients were placed in supine positions with the forearm in a semi-pronated (neutral) position. The following MRI protocols were adopted: axial fast spin-echo proton-density weighted image with fat saturation (repetition time (TR)/echo time (TE) 2300–3900/30–60 ms, slice thickness: 3 mm, interslice gap: 0 mm, field of view (FOV): 16 cm); oblique coronal fast spin-echo T2-weighted images with fat saturation (TR/TE: 2300–4600/30–50 ms; slice thickness: 2 mm; interslice gap: 0 mm; FOV: 16 cm); and sagittal fast spin-echo T2-weighted images with fat saturation (TR/TE: 2300–4600/30–50 ms; slice thickness: 3 mm; interslice gap: 0.3 mm; FOV: 16 cm).

Tendon integrity was categorized as follows according to Sugaya’s classification using T2-weighted oblique coronal MRI scans [19]. Type I indicated a repaired cuff that had sufficient thickness with homogeneously low intensity on each image. Type II indicated sufficient thickness associated with a partially high-intensity area, while type III had insufficient thickness without discontinuity. Type IV exhibited minor discontinuity in more than one slice of each image, thereby suggesting a small tear. Type V exhibited a major discontinuity in each image, thereby suggesting a medium or large tear [20]. The definition of retear refers to the Sugaya’s type IV and V. Retear pattern was classified into two types according to Rhee’s classification [21] on T2-weighted oblique sagittal scans: type 1, which indicates that the cuff tissue repaired at the rotator cuff insertion site was not observed to remain on the greater tuberosity, and type 2, which indicates that the remnant cuff tissue remained at the insertion site despite retear.

Fatty degeneration was evaluated for a torn muscle using the five-stage grading system presented by Goutallier et al. [22,23] on T_1_-weighted MRI scans. The tear size and level were measured on oblique coronal and oblique sagittal MRI views, respectively, because they increased the failure rate of repaired rotator cuffs [24]. All MRI results were evaluated by two authors. In the case of disagreements, they determined the respective grades via thorough discussions. The global fatty degeneration index (GFDI) is the mean value of Goutallier grades for supraspinatus, infraspinatus, and subscapularis [25].

### 2.5. Surgical Procedure and Rehabilitation

All surgical procedures were performed by a single surgeon who had over 20 years of experience in a single institution. The rotator cuff tear size was measured using an arthroscopic probe. Biceps tenotomy (*n* = 12) and tenodesis (*n* = 2) were performed for the dislocated or torn long head of the biceps tendon. In all cases, we also decompressed the bony spurs of the acromion using acromioplasty. The footprint of the torn rotator cuff was prepared using a motorized burr to debride soft tissue remnants and promote bone-tendon healing. Two medial row anchors (Y-knot RC all-suture anchor system, ConMed, Utica, NY, USA) were inserted at the medial border of the greater tuberosity footprint adjacent to the articular surface, and the lateral anchors were placed on the lateral aspect of the humerus relative to the shape of the bridge configuration. Regarding lateral row fixation, various knotless suture anchors were utilized. The patients underwent postoperative rehabilitation. One week after surgery, pendulum exercises were initiated. Active-assisted ROM exercise commenced 6 weeks after the operation. Three months after surgery, the patients were encouraged to start isometric muscle exercises with a rubber band. Minor sport activities were allowed after full ROM, and adequate muscle strength was obtained at 3 to 6 months after surgery according to the tear size [26].

We administered anti-osteoporotic drugs 2 days after surgery with close observation for side effects, such as fever, myalgia, arthralgia, and headache. If side effects occurred, acetaminophen tablets were administered for symptom alleviation. We intravenously administered 5 mg of zoledronic acid.

### 2.6. Statistics

We used SPSS version 26 (IBM SPSS, Chicago, IL, USA) to conduct all statistical analyses. Functional assessments were analyzed with the student t-test and paired t-test for comparisons between groups and comparisons in the same group with numerical data, respectively. χ^2^ and Fisher’s exact tests were applied for the categorical variables. To compare the retear rate between the groups, we used the non-inferiority (NI) test with a non-inferiority margin of 10% according to half of upper margin of the confidence interval in Oh’s study [27,28,29,30]. Statistical significance was set at *p* < 0.05.

## 3. Results

In total, 30 patients who were eligible for the study were enrolled. For the control group, 60 patients with normal BMD were enrolled via 1-to-2 matching using the propensity score. The demographic data of the two groups were not significantly different, except for BMD. Interestingly, both groups included only women. The degrees of preoperative fatty degeneration were similar in both groups (supraspinatus (*p* = 0.0.262), infraspinatus (*p* = 0. 769), and subscapularis (*p* =0.945)). The tear sizes measured by MRI in both groups were 31.9 ± 8.3 and 29.8 ± 13.3 (Table 1). The tear size of the tendons was negligibly larger in Group 1 than in Group 2.

### Functional and Radiographic Outcomes

In Group 1, the functional scores, which include SST, UCLA shoulder score, ASES, and CSS, improved significantly from baseline at 6 months after surgery. In Group 2, the functional scores, including SST, UCLA shoulder score, ASES, and CSS, improved from baseline at 6 months after surgery, and these improvements were statistically significant. The UCLA and CSS at 6 months were significantly higher in Group 2 compared with Group 1. The ASES score was significantly higher in Group 2 at both baseline and 6 months postoperatively. At 6 months after surgery, the ROM values between the two groups, including forward flexion, internal rotation, and external rotation, exhibited significant improvements. The UCLA score was significantly different between groups at 6 months postoperatively. According to the CSS, it also showed significant differences between groups at baseline and 6 months postoperatively. The retear rates were 13.3% (4 of 30 patients) and 25% (15 of 60 patients) in Groups 1 and 2, respectively. This showed the confidence interval as −0.05~0.29 using NI, indicating significance over an NI margin of 10%. The retear pattern in Group 1 was type I in all cases; however, three cases of type I and four cases of type II patterns were observed in Group 2, which had no statistical significance (Table 2).

## 4. Discussion

This is the first study to investigate the effect of an IV zoledronate on rotator cuff healing in human. The significant finding of the current study was that even patients with poor bone quality can achieve a reduction in failure rate, which exceeded the BMD of the normal population, provided we treat the patients with IV zoledronate. In a cohort study evaluating 272 patients who underwent rotator cuff repair, Chung et al. reported that the retear rate in patients with BMD < −2.5 was 41.7% and in patients with BMD > −2.5 was 13.2%. However, in the current study, Group 1 (osteoporosis with IV zoledronate injection) with tears over 2 cm in size with osteoporosis exhibited a lower failure rate than those reported in previous studies, and the results exceeded those of patients with normal bone density (13.3% vs. 25%, *p* = 0.172, CI; −0.05~0.29, ∆ = 10%) [2,3,5,31].

We assumed that zoledronate administration decreased the retear rate of repaired rotator cuff tendons by promoting bony ingrowth at the bone-to-tendon junction and by improving anchor stability. Bone ingrowth at the bone-to-tendon junction is required for preventing retear at the site. However, in osteoporotic patients, osteoclastic activity is excessive at the bone-to-tendon attachment site [32]. Rodeo et al. reported that the application of a receptor activator of nuclear factor-kappa B ligand (RANKL) impaired bone ingrowth to repaired tendons in a rabbit model. Contrarily, the administration of osteoprotegerin (OPG) significantly improved bone formation around the grafted tendon and improves the stiffness at the healing bone-to-tendon junction in the same model. Furthermore, bisphosphonate not only inhibits osteoclastic activity but also stimulates bone formation. Giuliani et al. reported that bisphosphonate promotes osteogenesis by stimulating osteoblast precursors and by promoting osteoblastogenesis in young and aged mice in vivo [33].

In their cadaveric study, Tingart et al. reported that the pull-out strength of suture anchors is closely related to bone quality. The time-zero strength of holding anchors by bone against pulling by repaired rotator cuffs is crucial in preventing anchor loosening, which may lead to a bone–tendon gap [34]. Schanda et al. also presented significant improvements in trabecular bone microarchitecture and biomechanical properties in the repaired tendon area after the chronic rotator cuff reconstruction with an adjuvant single-dose therapy of zoledronic acid in a rodent model compared with controls receiving saline injections [12]. In our case series, we had no anchor pull-out or dislocated cases. This may be due to the zoledronic acid injection, which compensated for the osteoclastic activity and promoted the stability of anchors in osteoporotic patients.

Owing to the properties of bisphosphonate, which interferes with bone turnover, concerns regarding its negative effects have been raised. Cancienne et al. reported the low effectiveness of bisphosphonate medications in osteoporotic patients in decreasing the failure rate after ARCR. Other studies have focused on the negative effects of using bisphosphonate, including cytotoxicity, impaired cellular proliferation, cellular migration, and wound healing, which impede and negatively affect rotator cuff healing [7,35,36]. However, the data analyses in these studies are based on a national database that could be miscoded and noncoded; therefore, the results obtained from these studies can be misleading. The bisphosphonate doses in these studies were excessively high to reflect the in vivo administration of bisphosphonate. Despite the debates on the negative effect of administering anti-osteoporotic drugs after ARCR, this study clearly demonstrated that, as a risk factor, osteoporosis can be offset by administering IV zoledronate. Although Sung et al. argued that a high concentration (100 × 10 ^−6^ M) of alendronate impaired the proliferation of fibroblasts and induced cell apoptosis in an in vitro study using human rotator cuff fibroblasts [36], the concentration in the study was 10 times higher than the therapeutic dose of alendronate sodium reported by Kum et al., such that osteoclast formation was significantly inhibited at the alendronate concentration of 10^−5^ M [37]. Considering that the mean oral bioavailability of alendronate in men and women was 0.59% and 0.64%, respectively, the in vivo concentration of daily oral ingestion of 10-mg alendronate was only 0.24 × 10^−6^ M. Although it appears that the injection of 5 mg of IV zoledronate with a monoisotopic mass of zoledronate at 272 g/mol induces high serum concentration, which negatively affects cells, the half-life of zoledronate is only 146 h, and it is washed out of the body. Hence, we can assume that a temporarily high serum concentration has a negligible effect on the cells compared with the in vitro environment. Moreover, the 10 times higher potency of zoledronate than that of alendronate allows for its single administration in a year, which causes less damage to the cells [38]. Our findings suggest that it is necessary to take advantage of administering IV zoledronate to patients to improve the integrity of the repaired tendon, regardless of the concerns related to the use of anti-osteoporotic drugs.

Interestingly, all of the patients enrolled in the current study were females aged > 60 years because only female patients met the inclusion criteria of low BMD < −2.5 and > 2 cm tear size. In addition, the national health insurance service prohibits prescribing DEXA for males aged under 70 unless they have suspicious pathologic fractures. Hence, we could not include male participants in our study. Older women are likely to have lower BMD than men owing to hormonal alterations after menopause [39,40]. Cadet et al. reported the effect of estrogen on the mechanical properties of rotator cuff tendons. They found that estrogen deficiency increases the stiffness and modulus of the supraspinatus tendon and that enthesis is a dynamic structure that responds to systemic hormonal changes [9]. We carefully assumed the retear rate of Group 2 (normal bone density) being relatively higher than that in Chung’s report [5] was because of aging and subsequent hormonal alteration. In our study, because we controlled for age and sex, we could not evaluate and conclude the effect of hormonal alteration in menopausal women; instead, we only evaluated the administration of anti-osteoporotic drugs and their effects. We carefully assume that using an estrogen agent concurrent with an anti-osteoporotic drug may be synergistically beneficial in minimizing the failure rate.

We found the UCLA and CSS at 6 months were significantly higher in Group 2 and that the ASES score was significantly higher in Group 2 either at baseline and 6 months postoperatively. Osteoporosis is highly associated with lower muscle mass. Additionally, lower muscle mass might affect the lower functional scores in Group 1 [41,42]. Szulc et al. stated that decreased muscle mass is associated with poor bone quality and with lower daily living activities. Therefore, we could assume that osteoporotic patients had less muscle mass and lower activity level than the control group. However, we could not elucidate which is the preceding cause: whether it is shoulder pain which led to lower activity or lower daily activity followed by sarcopenia.

Furthermore, this study had several limitations. First, owing to its small sample size, this study is limited in demonstrating the efficacy of zoledronate in patients with osteoporosis after ARCR. Further studies with larger sample sizes are required. Second, the most difficult factor designed in this current study was the designation of a control group, as BMD generally tends to decline with aging. It is evident that enrolling three or four times more people in a control group would help elicit more reliable statistical results. However, to match the control group with the study group, we had to settle for the 60 patients in the control group. As we treated all patients with BMD < −2.5 with various anti-osteoporotic drugs, no patient had osteoporosis without taking anti-osteoporotic drugs. In addition, as age increased, BMD tended to decrease simultaneously. Consequently, we could only perform 1-to-2 matching at most. A larger population is required for further studies. Third, we did not exclude patients who had traumatic events. This may mislead the results regarding pathologies by trauma and degeneration being different. However, the candidates had previous shoulder pain before a traumatic event (falling down). Moreover, all the patients were over 60s, which means that we could assume they had prior degenerative cuff lesions before falling down. Fourth, we did not include other systemic diseases as confounding factors. We had four retear cases in the osteoporotic group. Among the four cases, one had diabetes mellitus and dyslipidemia, one had hypothyroidism, and the other two had no underlying diseases (BMD: −4.0,−2.9,−4.2, and −2.8; tear size: 37.8 mm, 29.7 mm, 22.9 mm, and 41.3 mm, respectively). Two patients had no underling diseases, and the tear sizes were diverse. We could not find any distinctive factors affecting the result in a group. Diabetes mellitus, hyperlipidemia, and hypothyroidism are risk factors for rotator cuff disease. However, we did not structure the current study based on their underlying diseases [5,43]. Thus, it is required to include underlying diseases as risk factors in a further study. Finally, we did not perform histological analysis. Although the patients in the study group exhibited comparable outcomes with the control group, we could not determine any definite microscopic improvements in vivo. Performing further second-look surgeries on the patients would enable us to include histological findings in further studies.

In conclusion, the administration of IV zoledronate may reduce the failure rate after ARCR > 2 cm in length in older female patients with osteoporosis. Moreover, thorough scrutiny is required to detect osteoporosis in patients with rotator cuff tears.

## Figures and Tables

**Table 1:** Demographic data.

Variables	Group 1	Group 2	*p* Value
No. of patients	30	60	
Age, mean ± SD, year (range)	73.7 ± 4.9 (66–84)	72.0 ± 4.2 (64–84)	0.094
Sex, male/female, *n*	0/30	0/60	
Onset, mean ± SD, mo	13.9 ± 29.6	12.9 ± 23.0	0.865
Bone mineral density (T-score), mean ± SD	−3.6 ± 0.7	−0.4 ± 0.8	< 0.001 *
Preoperative fatty degeneration, mean ± SD			
Supraspinatus	2.3 ± 1.2	2.0 ± 1.5	0.262
Infraspinatus	1.0 ± 1.3	0.9 ± 1.2	0.767
Subscapularis	1.0 ± 1.0	1.0 ± 1.1	0.945
Global fatty degeneration index	1.4 ± 1.0	1.3 ± 1.1	0.521
Tear size, mean ± SD, mm	31.9 ± 8.3	29.8 ± 13.3	0.414
Smoking history, yes/no, *n*	0/30	0/60	
Trauma history, yes/no, *n*	8/22	20/40	0.632
Regular exercise, yes/no, *n*	27/3	52/8	0.746

NOTE. Data are presented as mean ± standard deviation (SD), or *n*. mo: month; *n*: number; SD: standard deviation. *: statistical significance.

**Table 2:** Comparison of clinical and radiographic outcomes between Groups 1 and 2.

Factors	Group 1	Group 2	*p* Value
Clinical Outcomes			
SST			
preoperative	4.4 ± 3.2	4.5 ± 2.4	0.855
postoperative at 6 months	6.3 ± 2.4	7.2 ± 2.4	0.109
*p*-value	0.004 *	<0.001 *	
UCLA			
preoperative	14.4 ± 6.3	15.4 ± 5.6	0.459
postoperative at 6 months	21.7 ± 6.6	25.3 ± 7.1	0.031 *
*p*-value	<0.001 *	<0.001 *	
ASES			
preoperative	44.6 ± 20.2	53.4 ± 17.6	0.039 *
postoperative at 6 months	68.4 ± 14.2	75.6 ± 13.7	0.034 *
*p*-value	<0.001 *	<0.001 *	
CSS			
preoperative	38.1 ± 17.8	42.9 ± 15.8	0.208
postoperative at 6 months	54.4 ± 17.5	65.8 ± 15.9	0.006 *
*p*-value	0.001 *	<0.001 *	
ROM			
Forward flexion			
preoperative	155.7 ± 34.8	145.8 ± 49.6	0.338
postoperative at 6 months	168.1 ± 25.4	167.8 ± 31.7	0.966
*p*-value	0.228	0.001 *	
Internal rotation			
preoperative	3.9 ± 4.3	4.6 ± 4.6	0.706
postoperative at 6 months	9.4 ± 5.9	8.6 ± 6.1	0.554
*p*-value	0.003 *	<0.001 *	
Radiographic outcomes			
Retear, % (*n*/*N*, 95%CI)	13.3 (4/30)	25 (15/60, −0.05~0.29 *)	0.172
Retear pattern, *n*, type I/II	4/0	8/7	0.088

NOTE. Data are presented as mean ± SD unless otherwise noted. To measure the internal rotation, the level of the vertebra was numbered serially from the 0 point (below L5) to 14 points (at T4 level). The non-inferiority margin (∆) was set as 10%. *: statistical significance; SST: simple shoulder test; UCLA: UCLA shoulder score; ASES: American Shoulder and Elbow Surgeons; CSS: constant shoulder score; 95% CI: 95% confidence interval.
